# Introgressive hybridization erodes morphological divergence between lentic and lotic habitats in an endangered minnow

**DOI:** 10.1002/ece3.8086

**Published:** 2021-09-15

**Authors:** Henry K. Baker, Danielle C. Hankins, Jonathan B. Shurin

**Affiliations:** ^1^ Section of Ecology, Behavior, and Evolution University of California San Diego La Jolla CA USA

**Keywords:** body shape evolution, cyprinid, divergent selection, hybrid swarm, introgression, local adaptation

## Abstract

Introgressive hybridization may erode phenotypic divergence along environmental gradients, collapsing locally adapted populations into a hybrid swarm. Alternatively, introgression may promote phenotypic divergence by providing variation on which natural selection can act. In freshwater fishes, water flow often selects for divergent morphological traits in lake versus stream habitats. We tested the effects of introgression on lake–stream morphological divergence in the minnow Owens Tui Chub (*Siphateles bicolor snyderi*), which has been rendered endangered by introgession from the introduced Lahontan Tui Chub (*Siphateles bicolor obesa*). Using geometric morphometric analysis of 457 individual Tui Chub from thirteen populations, we found that both native and introgressing parent taxa exhibited divergent body and caudal fin shapes in lake versus stream habitats, but their trajectories of divergence were distinct. In contrast, introgressed populations exhibited intermediate body and caudal fin shapes that were not differentiated by habitat type, indicating that introgression has eroded phenotypic divergence along the lentic–lotic gradient throughout the historic range of the Owens Tui Chub. Individuals within hybrid populations were less morphologically variable than those within parent populations, suggesting hybrid adaptation to selective agents other than water flow or loss of variance by drift.

## Graphical abstract

Introgressive hybridization can either erode or promote adaptive phenotypic divergence along environmental gradients. We tested the effects of widespread introgressive hybridization on morphological divergence between lake and stream habitats in the minnow Tui Chub. We found that hybridization has eroded morphological divergence, but comparisons of morphological variance between parent and hybrid populations suggest this outcome may not be maladaptive.

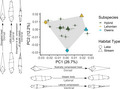

## 1 INTRODUCTION

Populations occupying different habitats experience distinct selective pressures. The extent to which these populations diverge phenotypically to meet the demands of their local environment affects population fitness and can substantially alter ecological processes (Bassar et al., 2010; Hairston et al., 2005; Palkovacs & Post, 2009; Post & Palkovacs, 2009; Yoshida et al., 2003). Introgressive hybridization, the incorporation of genetic material from one species or subspecies into another, can alter evolutionary trajectories in ways that either promote or degrade divergence along selective gradients, but empirical tests are needed to determine which outcomes prevail in nature (Abbott et al., 2013; Allendorf et al., 2001; Todesco et al., 2016).

The degree of phenotypic divergence across an environmental gradient depends on the interactive effects of selection, gene flow, and phenotypic plasticity (Crispo, 2008; Hendry et al., 2001, 2002; Hendry & Taylor, 2004). Strong divergent selection imposed by environmental gradients promotes adaptive phenotypic divergence (i.e., local adaptation) (Schluter, 2001), while gene flow across disparate habitats erodes it (Slatkin, 1987). Phenotypic plasticity can promote either adaptive or maladaptive phenotypic divergence (Ghalambor et al., 2007), though maladaptive phenotype–environment correlations produced by plasticity are often muted by countergradient genetic variation (Conover & Schultz, 1995; Urban et al., 2020). Similar to gene flow within a metapopulation, gene flow from external sources via introgressive hybridization may inhibit adaptation by collapsing locally adapted populations into a hybrid swarm (Allendorf et al., 2001; Rhymer & Simberloff, 1996; Todesco et al., 2016). Alternatively, the introduction of genetic variation by introgressive hybridization may promote adaptation by purging deleterious mutations, allowing selection to act on previously fixed but maladaptive traits, or producing novel adaptive phenotypes (Abbott et al., 2013; Lewontin & Birch, 1966; Seehausen, 2004; Selz & Seehausen, 2019).

In freshwater fishes occupying lake–stream networks, water flow generates divergent selection on morphological traits (Brian Langerhans & Reznick, 2010; Hubbs, 1940; Langerhans, 2008), and phenotypic divergence between lake and stream populations has been documented in a variety of fish taxa (e.g., Bolnick & Paull, 2009; Brinsmead, 2003; Collin & Fumagalli, 2011; Langerhans et al., 2007; Mcguigan et al., 2003; McLaughlin & Grant, 1994). Meta‐analysis has shown not only that lake–stream divergence is common but also that trait differences between habitat types are consistent across taxa and match predictions from biomechanical models of swimming performance in flowing versus still water. Parallel evolution among independent lineages suggests that these patterns are adaptive (Brian Langerhans & Reznick, 2010; Langerhans, 2008).

In the Owens River basin on the eastern slope of the Sierra Nevada Mountains (California, USA), the minnow Owens Tui Chub (*Siphateles bicolor snyderi*) was historically abundant in a variety of lentic and lotic habitats (Miller, 1973; Snyder, 1917). The Owens Tui Chub is thought to be derived from an ancestral population that occupied an interconnected network of massive lakes during the Pleistocene that included the Lake Lahontan drainage to the north and the Death Valley system to the south (Miller, 1946). Owens Tui Chub diverged from the Lahontan Tui Chub (*Siphateles bicolor obesa*) following geographic isolation which has been maintained since at least the late Pleistocene (Reheis et al., 2002). By the 1960s, however, Lahontan Tui Chub had been introduced to the Owens Basin, presumably by recreational fishermen using them as bait for non‐native trout (Miller, 1973). The introduced Lahontan and native Owens Tui Chubs readily hybridized and Lahontan alleles rapidly introgressed throughout nearly the entire historic range of the Owens Tui Chub (Chen et al., 2007; Miller, 1973). This widespread introgression rendered the Owens Tui Chub endangered by 1985, and they now persist in only six known isolated populations (USFWS, 1998, 2009). Robust hybrid populations now occupy lakes and streams throughout the Owens River basin (Chen et al., 2007). The phenotypic consequences of this rapid introgression and its effects on population fitness are largely unknown (but see Galicia et al., 2015; Leunda et al., 2013).

**FIGURE 1:**
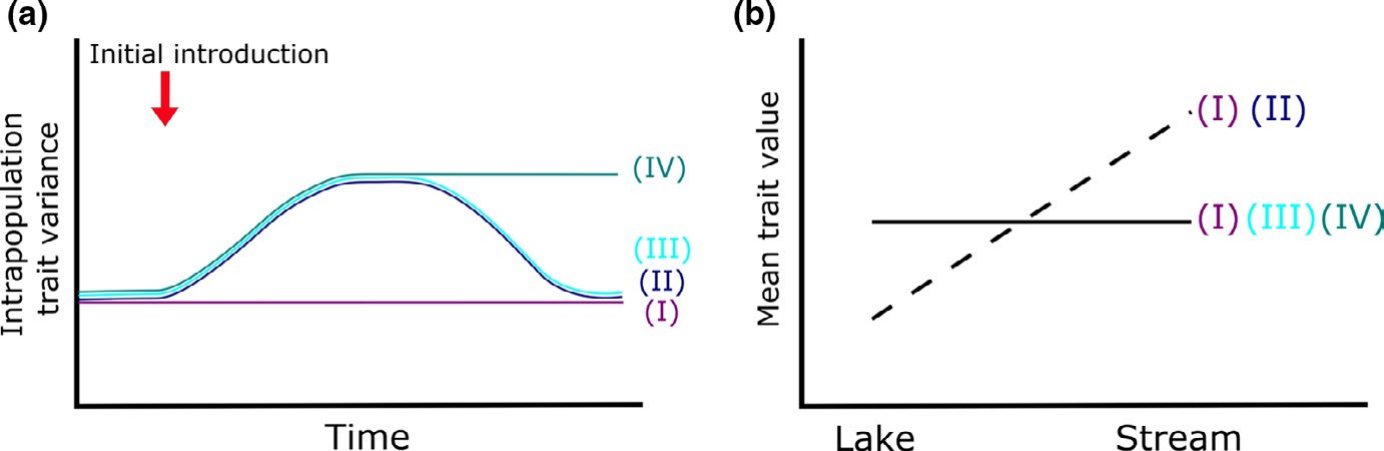
Conceptual model of the potential outcomes of introgression on intrapopulation trait variance (a) and mean trait values in lake versus stream habitats (b). Scenario (I) shows the absence of introgression (or strong selection against introduced individuals), where trait variance remains constant and lake–stream trait divergence depends on initial conditions. Scenario (II) shows an increase in trait variance following introgression and subsequent reduction by natural selection, producing divergent traits in lake versus streams. In Scenario (III), trait variance follows the same time course as (II), but variance reduction proceeds by drift (or selection by factors other than flow), so populations do not diverge phenotypically along the lake–stream axis. In Scenario (IV), trait variance increases following introgression but remains high due to continued gene flow overwhelming selection (genetic swamping) or relaxed divergent selection, producing populations that do not differentiate along the lake–stream axis

Here, we evaluate the effects of introgression on morphological divergence between lake and stream habitats in Tui Chub. Figure 1 shows the potential outcomes of introgression on intrapopulation trait variance and mean trait values in lake versus stream habitats. In Scenario I, no introgression takes place, because either no fish are introduced or strong selection acts against introduced phenotypes; in this case, trait variance is constant and divergence between lake and stream habitats is maintained if it was already present. Widespread introgression has already been documented in our system (Chen et al., 2007), so Scenario 1 represents the non‐introgressed parent populations. With introgression, trait variance increases due to reshuffling of parental genomes (Scenarios II–IV). If continued gene flow is limited, we expect trait variance to decrease over time, either adaptively via selection or randomly via drift, with lake–stream divergence expected in the former but not the latter (Scenarios II and III, respectively).

To evaluate this range of potential outcomes of introgression in Tui Chub, we sampled 457 fish from thirteen populations (nine hybrids, two Owens, and two Lahontan). We used geometric morphometrics of body shape from three perspectives (dorsal, lateral, and ventral) and measured caudal fin aspect ratio to test whether Owens, Lahontan, and hybrid Tui Chub exhibit morphological divergence between lake and stream habitats and compared the trajectory and magnitude of phenotypic divergence in the two parental taxa and their hybrids. We then evaluated the effects of introgression on intrapopulation morphological variation by comparing hybrid and parental populations.

## 2 METHODS

### 2.1 Fish sampling

We collected fish in July and August in 2019 and 2020, except for one site (East Walker River) which we sampled in February 2021, using beach seines and backpack electrofishing. We sampled one lake and one stream population from each of the parent subspecies (Owens and Lahontan), and four lake and five stream populations of putatively introgressed populations (see Appendix S1 for site descriptions). We sampled a total of 457 individual Tui Chub (mean = 35 fish/site). For the Lahontan and hybrid populations, we humanely euthanized the fish upon collection according to UCSD IACUC Protocol #S14140. For the endangered Owens populations, we took photographs and measurements of live anesthetized fish, then revived and released them. We measured all fish to the nearest millimeter (standard length), weighed to the nearest centigram (wet weight), and photographed from three perspectives (dorsal, ventral, and lateral) for morphometric analysis.

### 2.2 Morphometric analysis

**FIGURE 2:**
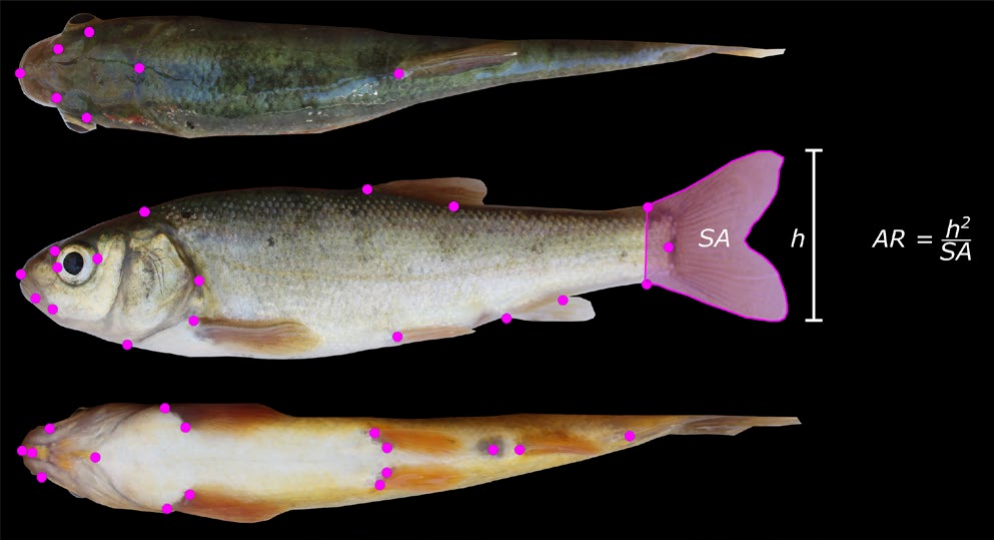
Homologous landmarks used for geometric morphometrics of Owens, Lahontan, and hybrid Tui Chubs. Shown above is an Owens Tui Chub (SL = 103mm). We used seven dorsal (top), eighteen lateral (middle), and sixteen ventral landmarks (bottom) [see Armbruster (2012) for anatomical descriptions of landmark locations]. Pink shaded region shows caudal fin surface area used for calculating caudal fin aspect ratio

For each fish, we placed sixteen ventral, eighteen lateral, and seven dorsal homologous landmarks (Armbruster, 2012) using the R package “StereoMorph” (Olsen & Westneat, 2015) (Figure 2). A single researcher placed all landmarks to avoid potentially confounding effects. We performed generalized procrustes analysis (GPA) to align and adjust the raw landmarks, providing centroid size and shape coordinates (Gower, 1975; Rohlf & Slice, 1990) using the R package “geomorph” (Adams et al., 2020). Sexual dimorphism has not been reported in Tui Chub, so we did not perform separate morphometric analyses by sex. To calculate caudal fin aspect ratio, we digitally measured caudal fin height and surface area using the software “ImageJ” (Schneider et al., 2012) (Figure 2). We calculated caudal fin aspect ratio (AR) as the squared height of the fin divided by the surface area: AR = h^2^/SA (Brian Langerhans & Reznick, 2010).

### 2.3 Statistical analysis

We evaluated differences in body shape between introgressed populations from lake versus stream habitats using linear models of procrustes residuals with centroid size (log‐transformed) as a fixed covariate to account for allometry, habitat type (lake or stream) as a fixed effect, and site as a nested random effect using the “procD.lm” function in the R package “geomorph” (Adams et al., 2020). In order to evaluate the effect of habitat type relative to the variation among the sites (which we “randomly” sampled from the set of possible sites), we used the mean squares of the interaction term (habitat type:site) as the denominator for calculating the *F*‐statistic for habitat type. We modeled each of the three perspectives (dorsal, lateral, and ventral) separately to avoid potential scaling issues associated with allometric models of combined landmark sets (Collyer et al., 2020). To evaluate divergence between the lake and stream populations for each of the parent subspecies, we used fixed effects models with centroid size (log‐transformed), habitat type, subspecies, and a subspecies:habitat type interaction term. For all models described above, we tested for significance of independent variables using randomized resampling permutation procedures (Collyer & Adams, 2018).

We analyzed the effects of habitat type on caudal fin aspect ratio for the introgressed populations using linear mixed models fit with the R package “lme4” and evaluated for significance using the “lmerTest” package (Bates et al., 2015; Kuznetsova et al., 2017). We used the fish standard length (log‐transformed) as a fixed covariate to account for allometry, habitat type as a fixed effect, and site as a random effect. We modeled the parent populations separately using linear models with standard length (log‐transformed) as fixed covariate, habitat type and subspecies as fixed effects, and a habitat:subspecies interaction term. For visualizing the allometry‐free effects of habitat type and taxonomic identity on caudal fin aspect ratios, we used the residuals of linear allometric models as the dependent variable. We estimated within‐population morphological disparity (also called morphological variance) as procrustes variance and performed pairwise comparisons via permutation using the “morphol.disparity” function from the “geomorph” package (Collyer et al., 2020).

## 3 RESULTS

### 3.1 Body shape

**FIGURE 3:**
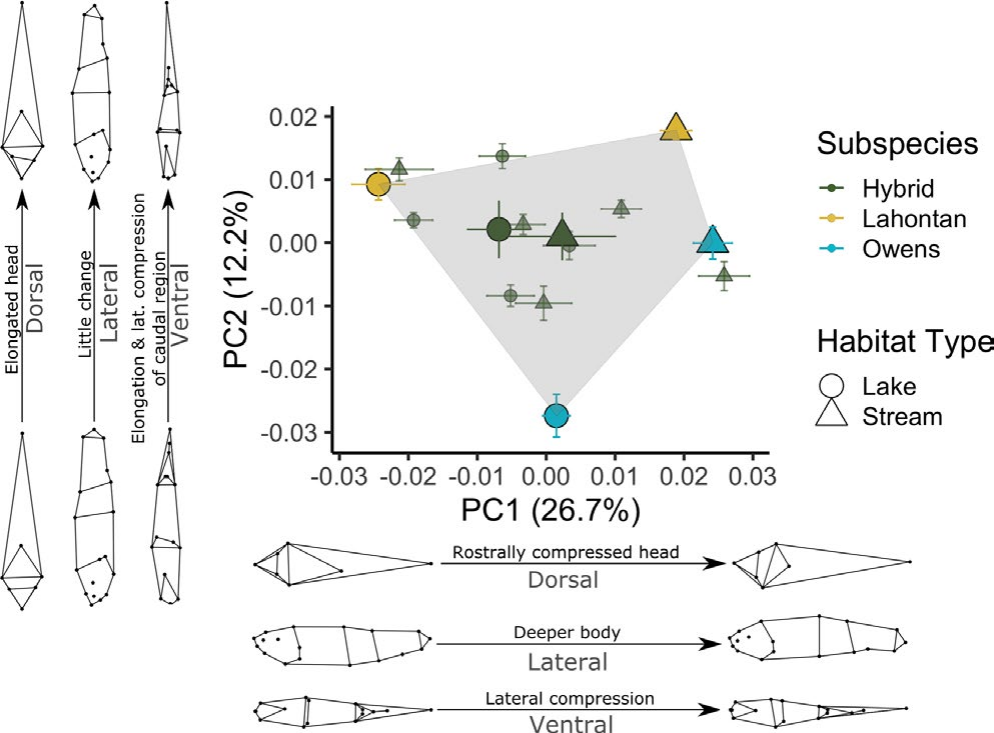
Ordination of allometry‐free GPA‐aligned landmarks for all three (dorsal, lateral, and ventral) views by principal component analysis. Points represent population means (± *SE*), except for large green points which show mean and standard error across hybrid populations (small green points). Shape denotes whether populations occupy lake (circles) or stream (triangles) habitats and color denotes putative subspecies. Gray shading shows the convex hull of parent subspecies means. Point diagrams show body shape for individuals representing the minimum and maximum points along each principal axis. Percentages on axis titles denote the percent of total variance in body shape contained on that axis

**FIGURE 4:**
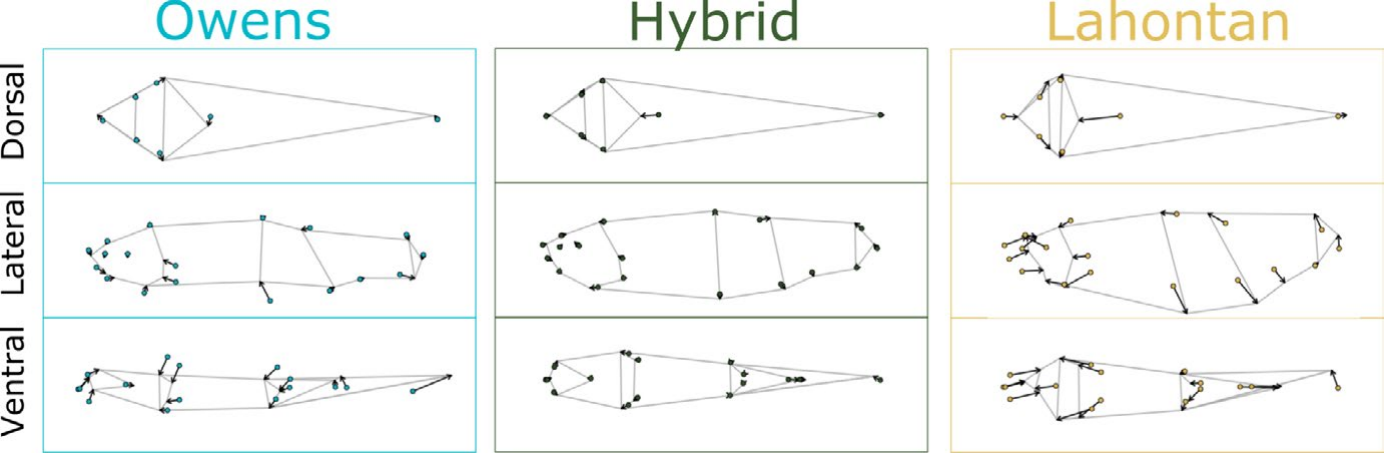
Body shape divergence between Tui Chub populations from lake (points) and stream (gray outline, vector destinations) habitats from dorsal (top), lateral (middle), and ventral (bottom) perspectives. Vectors show change in allometry‐free GPA‐aligned landmarks from the mean lake phenotype to the mean stream phenotype (magnified 3x to highlight changes)

Both parental subspecies of Tui Chub (Lahontan and Owens) exhibited body shape divergence between lake and stream populations for all three perspectives (ventral, lateral, and dorsal: *p* < .001 for all), though the magnitude of divergence was greater for Lahontan Tui Chub (*p* < .01 for all habitat type:subspecies interactions, Tables S2‐1–S2‐3 in Appendix S2). Principal component analysis of allometry‐free GPA‐aligned landmarks for all three perspectives combined (i.e., dorsal, lateral, and ventral) produced two principal axes that contained 39% of total variance in body shape (Figure 3). For Lahontan Tui Chub, lake–stream divergence occurred primarily along PC1, while Owens Tui Chub exhibited greater divergence along PC2, reflecting distinct trajectories of trait divergence (Figure 3). Lahontan Tui Chub from stream populations had deeper bodies and caudal peduncles, rostrally compressed and broader head regions, and shortened caudal regions relative to lake populations (Figure 4). Lake–stream morphological differences were less pronounced and were reflected in different shape aspects in Owens Tui Chub: relative to the lake population, mean body shape in the stream population was more laterally compressed with elongated caudal regions (Figure 4).

Introgressed populations exhibited intermediate body shapes relative to the parental subspecies: Six of nine (67%) population means were bounded within the convex hull connecting the four parental population means. Linear models provided no evidence for lake–stream divergence in body shape for the introgressed populations (main effect of habitat type: *p* > .05 for all three perspectives; Tables S2‐1–S2‐3).

### 3.2 Caudal fin aspect ratio

**FIGURE 5:**
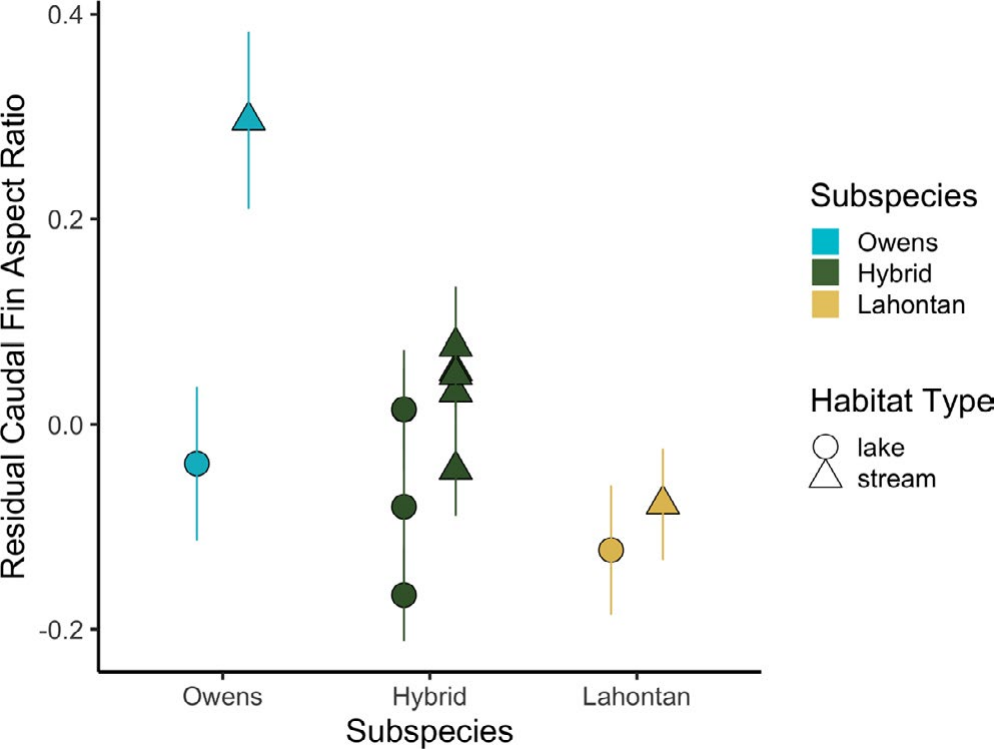
Effects of introgression and habitat type on caudal fin aspect ratio. Points show population mean (± *SE*) residual caudal fin aspect ratio after removing allometric effects via linear model of aspect ratio versus standard length (log‐transformed). Color and shape denote subspecies and habitat type, respectively

For parental subspecies, caudal fin aspect ratio was higher in stream than lake habitats and was higher for Owens than Lahontan Tui Chub (*p* < .01; Figure 5, Table S2‐4). Allometry (fish standard length) had a marginally insignificant effect on caudal fin aspect ratio (*p* = .051). In the introgressed populations, only fish length affected caudal fin aspect ratio (*p* << .001) (Table S2‐4).

### 3.3 Morphological disparity

**FIGURE 6:**
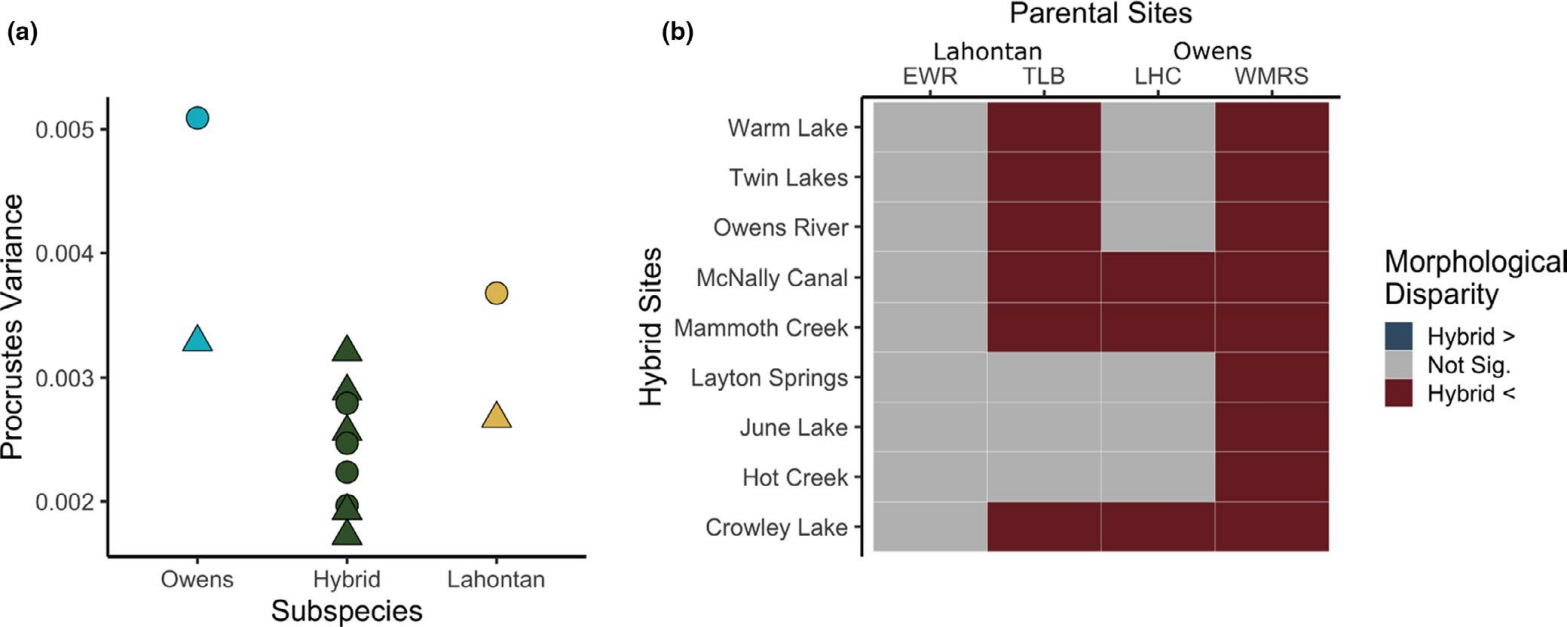
Comparison of within‐population morphological disparity. (a) Procrustes variances for each population by subspecies, with stream populations shown as triangles and lake populations as circles. (b) Results of pairwise comparisons of morphological disparity between hybrid populations and parent subspecies populations performed with permutation tests (α = 0.05). Blue or red indicate the hybrid site was significantly more or less variable than the parent population, respectively

Morphological disparity within populations was highest in the lake populations of the parent subspecies (Figure 6; Tables S2‐5 and S2‐6). Hybrid populations had relatively low morphological disparity that did not vary by habitat type; in pairwise comparisons of hybrid populations to parent populations, no hybrid population was more variable than any of the parent populations (Figure 6; Table S2‐6).

## 4 DISCUSSION

Introgressive hybridization may erode phenotypic divergence along environmental gradients, collapsing locally adapted populations into a hybrid swarm. Alternatively, introgression may promote divergence by providing variation on which natural selection can act. Our results indicate that hybrid Tui Chub are intermediate to the parental subspecies in morphology and exhibit less divergence between lake and stream habitats. This result indicates that hybridization may impede adaptation to divergent selection imposed by lotic versus lentic environments, although the hybrids may differ from the parent species in terms of other ecological functions or characteristics.

The implications of this finding for local adaptation and population fitness rest in part on which point we sampled along the time course of hybridization‐induced evolutionary change. Gene flow from distinct taxa via introgression is at first a variance‐generating process that produces a suite of phenotypes with variable fitness (e.g., hybrid swarms) due to recombination between parental genomes (see Figure 1a; Grant & Grant, 1994; Stebbins, 1959). If we sampled during this initial stage, we would expect higher intrapopulation morphological variance in the introgressed relative to parental populations and low divergence across ecological gradients, regardless of whether introgression ultimately proved adaptive or maladaptive. Following the initial flux, with limited continuing gene flow, we would expect intrapopulation phenotypic variance to decline over time, either adaptively via selection or randomly via drift (Figure 1a). If the observed absence of lake–stream morphological divergence was a transient effect preceding the action of divergent selection or drift, we would expect high intrapopulation morphological variance in introgressed relative to the parent populations. Instead, we found that individuals in introgressed populations showed more homogenous morphologies relative to the parental populations: Pairwise comparisons of intrapopulation morphological disparity showed that none of the nine introgressed populations we sampled were significantly more variable than any of the four parent populations. In fact, hybrid populations exhibited significantly lower variance than parental populations in 50% of pairwise comparisons. This suggests that natural selection or drift has reduced the variance initially introduced by introgression, leaving reduced substrate for the action of future divergent selection by water flow.

The low intrapopulation morphological variance in introgressed populations also undermines genetic swamping (Scenario IV in Figure 1)—where gene flow overwhelms selection, resulting in the loss of locally adaptive alleles—as an explanation for the pervasive introgression throughout the historical range of the Owens Tui Chub. Genetic swamping is the most commonly reported mechanism of hybridization‐driven extinction risk and is particularly common in hybridizing fishes (Todesco et al., 2016). However, if there were ongoing and widespread introductions of Lahontan Tui Chub, we would expect morphological variance in introgressed populations to remain high relative to parental populations due to continuing introduction of novel genotypes. Our data did not support this expectation. Genetic swamping is also inconsistent with the putative mechanism of introduction—use and subsequent release of Lahontan Tui Chub as live bait by trout fishermen in the Owens Basin (Miller, 1973). While Miller (1973) did not provide direct evidence for introduction by fishermen, there are no clear alternate explanations and geologic data indicate that the two basins have been geographically isolated since at least the late Pleistocene (Reheis et al., 2002).

The putatively small pool of introduced Lahontan Tui Chub paired with the rapid introgression throughout all connected bodies of water in the Owens Basin (Chen et al., 2007; Miller, 1973) is indicative of a selective sweep. However, in the context of biomechanical performance in flowing versus still water, the degradation of lake–stream morphological divergence in introgressed populations appears maladaptive. There are well‐documented trade‐offs in performance between steady (prolonged, straight‐line) and unsteady (e.g., fast‐start, change of direction, braking) swimming in fishes due to morphology, with the former favoring terete body forms with narrow caudal peduncles and high aspect ratio caudal fins, and the latter favoring deeper bodies and caudal regions with low aspect ratio, paddle‐like caudal fins (Brian Langerhans & Reznick, 2010; Webb, 1984). This trade‐off explains strong morphology–ecology correlations across fishes (Webb, 1984), including the prevalence of lentic–lotic ecomorphotypic divergence, as flowing environments require steady swimming to hold position (Brian Langerhans & Reznick, 2010; Langerhans, 2008). Indeed meta‐analysis has shown that high‐flow versus low‐flow morphological divergence is commonplace and generally consistent across fish taxa (Brian Langerhans & Reznick, 2010; Langerhans, 2008). In our study, the non‐introgressed Owens Tui Chub populations most closely matched the predictions for lake–stream divergence based on biomechanical principles: stream fish showed more streamlined, shallower bodies with narrow, elongated caudal peduncles and high caudal fin aspect ratios. In contrast, Lahontan Tui Chub from the stream population exhibited deeper bodies and shortened, deeper caudal peduncles, and only marginally higher aspect ratio caudal fins relative to lake populations.

The absence of expected lake–stream morphological differences in the introgressed Tui Chub may reflect adaptation to selective pressures other than water flow. Deviations from expectations based on water flow alone are not uncommon in the literature (e.g., Hendry et al., 2006; McGuigan et al., 2003; Neat et al., 2003), and morphology interacts with a number of environmental and ecological demands that affect fish fitness. Despite low intrapopulation variation in body shape, introgressed Tui Chub varied considerably across populations, consistent with prior work on bone morphology in hybrid Tui Chub (Galicia et al., 2015). This interpopulation variation perhaps reflects the wide range of ecological and environmental conditions encapsulated within our “lake” and “stream” categories (Appendix S1). “Stream” included populations from large rivers and their tributaries, spring fed streams, and slowly flowing irrigation canals, while “lake” included cold and clear montane lakes, a turbid reservoir, and a shallow alkaline lake. Food resources, water quality, habitat complexity, the magnitude and nature of predation risk, and other factors all interactively affect the direction and strength of selection (Brian Langerhans & Reznick, 2010; Langerhans et al., 2007). Predation risk, for example, tends to select for morphological traits that support unsteady swimming movements (Langerhans & DeWitt, 2004). In our study, Tui Chub were found in areas with dense aquatic vegetation in most of the populations we surveyed. The need to navigate complex habitats like weed beds favors morphologies compatible with unsteady swimming behaviors (Brian Langerhans & Reznick, 2010; Langerhans, 2008). In stream habitats, the aquatic vegetation may reduce the strength of current within the microhabitat that the fish occupy, thereby reducing the strength of selection on steady swimming capabilities. The combined effect of multiple competing demands determines a population's position along the trade‐off curve between steady and unsteady swimming (Brian Langerhans & Reznick, 2010). Thus, whether the erosion of lake–stream divergence in introgressed Tui Chub is maladaptive or epiphenomenal of some other adaptive process cannot be resolved from our data. Future work using comparative studies and experiments across a range of ecological and environmental conditions is required to determine the fitness consequences of the morphological outcomes introgression that we document here.

Three of nine introgressed populations we sampled exhibited principal component positions that fell outside the convex hull of the parental populations. While this may reflect novel, transgressive traits generated by introgression, we urge caution in this interpretation as our sampling design did not capture the full range of variation across populations of the parental subspecies. For non‐introgressed Owens Tui Chub, our sampling was limited by the endangered status of the taxa and was undertaken opportunistically during conservation action by state wildlife officials. Our stream sample captured the only known extant non‐introgressed stream population of the Owens Tui Chub. Prior to introgression, the Owens Tui Chub historically occupied more swiftly flowing environments, so the differences in water flow between lentic and lotic environments of the non‐introgressed populations we sampled were reduced compared to the pre‐introgression state. Thus, the lake–stream divergence in non‐introgressed Owens Tui Chub we measured likely underestimated the true magnitude of divergence across all pre‐introgressed populations.

## 5 CONCLUSIONS

Our study showed that introgression has eroded morphological divergence between lake and stream habitats in populations of the now endangered Owens Tui Chub. Low morphological variance within the introgressed populations provides little substrate for the actions of future divergent selection, indicating that introgression has reduced the capacity of Tui Chub to adapt to the divergent demands of flowing versus still water. Variation across populations may, however, reflect adaptation driven by other selective agents.

## CONFLICT OF INTEREST

The authors have no conflict of interest to declare.

## AUTHOR CONTRIBUTIONS

**Henry K. Baker:** Conceptualization (lead); Data curation (lead); Formal analysis (lead); Funding acquisition (lead); Investigation (lead); Methodology (lead); Project administration (lead); Resources (supporting); Supervision (supporting); Visualization (lead); Writing‐original draft (lead); Writing‐review & editing (equal). **Danielle C. Hankins:** Data curation (supporting); Investigation (supporting); Writing‐review & editing (supporting). **Jonathan B. Shurin:** Funding acquisition (supporting); Resources (lead); Supervision (lead); Writing‐review & editing (equal).

## Supporting information

Appendix S1

Appendix S2

## Data Availability

Data are available at https://doi.org/10.5061/dryad.dr7sqv9zt.
