# Supplementary material for: Introgressive hybridization erodes morphological divergence between lentic and lotic habitats in an endangered minnow
Source: Ecol Evol. 2021 Sep 15;11(19):13593–600. doi: 10.1002/ece3.8086 (PMC8495819; doi:10.1002/ece3.8086)
Supplement: Supplementary file 1 — Appendix S1 [file ECE3-11-13593-s002.docx]

**Appendix S1**

**Site Descriptions**

Twin Lakes Bridgeport— Lahontan, lake

Twin Lakes Bridgeport consists of two montane lakes connected by a short stream. The lakes  are framed by towering peaks and are fed by snowmelt near the town of Bridgeport in Mono County, CA. The lakes support non-native game species including stocked Brown Trout and Rainbow Trout, which provide a recreational fishery that has produced state-record sized fish. The water is cold and clear. Tui Chub tend to congregate in macrophyte beds, including near the marina on the upper lake. Lahontan redside are also native to the lakes. We sampled the upper of the two lakes. The lakes are drained by Robinson Creek, which flows to Bridgeport Reservoir.

East Walker River— Lahontan, stream

The East Walker River is fed by snowpack at its headwaters on the eastern slope of the Sierra Nevada in Mono County, CA. From its headwaters it passes through Bridgeport Reservoir which regulates its flow into Nevada, where it connects with the West Walker River before draining into the endorheic Walker Lake basin. The East Walker River constitutes one of the most southern portions of the Lahontan Tui Chub’s native range and as such is one of the geographically nearest populations to the Owens Tui Chub’s native range and a likely source pool for introductions by bait fishermen. We sampled Tui Chub from the tailwaters below Bridgeport Reservoir in February, 2021.

This section of the river is high gradient and swiftly flowing with cobble bottom punctuated by boulders. There is little aquatic vegetation and introduced predators, predominantly brown trout (*Salmo trutta*) and rainbow trout (*Oncorhynchus mykiss*), are abundant. As such, this habitat is generally hostile for Tui Chub, which are a structure-oriented prey fish reliant on aquatic vegetation for cover, and which typically are found in slow-flowing water. While we did not conduct a formal survey, we found only large adult Tui Chub, suggesting little in-river recruitment. Thus, the East Walker River Lahontan Tui Chub that we sampled may in fact be colonists from upstream Bridgeport Reservoir where the population likely experienced “lake” type conditions for several generations following the reservoir’s formation in 1923. Nonetheless, the population we sampled likely underwent at least one iteration of selection on body and fin shape traits by flowing water, as they experience severe flow events in the river— at times exceeding 800cfs in a confined channel— that may result in the mortality of poor swimmers. Prior research evaluated bone morphometry of four populations of Lahontan Tui Chub: East Walker River, Twin Lakes (Bridgeport), Walker Lake, and Lake Tahoe (Galicia et al. 2015). Clustering from discriminant analysis showed that the three lake populations were most similar morphologically, with the East Walker River as an outgroup to the other three despite sharing a watershed with Twin Lakes (Bridgeport). This is consistent with the notion that morphometry of fish in the East Walker River is in fact a “stream” population despite likely dispersal from Bridgeport Reservoir.

Little Hot Creek— Owens, stream

Little Hot Creek is a thermal spring-fed Creek in Long Valley in Mono County, California. The stream was artificially impounded in 1986 by the US Forest Service to create waterfowl habitat. Downstream of the impoundment is a series of potholes ~1-3m in diameter connected by subterranean flow and intermittent surface flow. The Owens Tui Chub population in Little Hot Creek was founded by the transplant of 811 individuals from Hot Creek Springs and Upper Owens Gorge in 1988. We sampled Tui Chub from the pothole habitats using a beach seine. Tui Chub appear to be the only fish species in the pothole habitats of the creek.

White Mountain Research Station Ponds— Owens, lake

There are three artificial ponds fed by pumped well water on the University of California’s White Mountain Research Station in the town of Bishop in Inyo County, CA. Two of these ponds contain an Owens Tui Chub population founded from the rescue of twenty two non-introgressed individuals, surprisingly discovered in the Lower Owens River Gorge after rewatering in 1997 (Chen et al. 2007).  The source population was subsequently extirpated, an event which has been attributed to the non-native Brown Trout population (Chen et al. 2007). The artificial ponds are shallow and consist of a tarp bottom (to prevent seepage) covered in an accumulated silty/organic substrate. The pond includes many tires laid flat to provide structure and refugke. Tui Chub are the only fish in the two ponds they occupy.

McNally Canal— Introgressed, stream

McNally Canal is a man-made network of channels for water conveyance from the Owens River and pumped groundwater sources to agricultural users in Inyo County, CA. The canals are slow-flowing with sand or silt substrate and steep banks. Tui Chub are found in portions of the canal that support dense beds of aquatic vegetation. Other fish species include non-native Common Carp and Largemouth Bass, as well as the regionally native Owens Sucker.

Owens River— Introgressed, stream

We sampled the upper Owens River, upstream of Lake Crowley. This section of the river meanders through a meadow that is grazed by cattle throughout the spring and summer. The channel braids at several points, forming narrower side channel habitats that tend to have slower flow than the main channel. Banks are steep and deeply undercut. The substrate is predominantly silt and sand. Aquatic vegetation is well established, especially in the side channel habitat. Tui Chub are primarily found amongst the stands of vegetation in both the side and main channel habitats. Besides Tui Chub, the native Owens Sucker is common in the river, as are stocked and wild-reproducing Brown and Rainbow Trout. Non-native threespine stickleback are common in portions of the river as well.

Hot Creek— Introgressed, stream

Hot Creek is a first order tributary to the upper Owens River near the town of Mammoth Lakes in Inyo County, CA. It is fed by Mammoth Creek as well as both cold and hot springs. As the name implies, the creek is substantially warmed by geothermal inputs and is much warmer than both Mammoth Creek upstream and the Owens River downstream. Downstream of the canyon where most geothermal activity occurs is a weir which is a barrier to upstream dispersal. Downstream of the weir, the creek becomes meanders through a grazed meadow before flowing into the Owens River. We sampled the stretch between the Owens River and the weir, which is densely vegetated across the majority of the channel. Tui Chub are common in the vegetated areas. Non-native mosquitofish are abundant throughout the creek.

Mammoth Creek— Introgressed, stream

Mammoth Creek is a coldwater stream that is a first order tributary to Hot Creek. This stream flows out of Twin Lakes (Mammoth) through a high gradient canyon section where introduced trout are abundant, but Tui Chub are rare or absent. It flows through the town of Mammoth Lakes and eventually connects with a spring-fed stream near the Hot Creek Trout Hatchery to form Hot Creek. Tui Chub appear to be abundant only in the lower reaches of Mammoth Creek, where it meanders through a grazed meadow. Here, the stream has predominantly silt substrate with some sparse stands of aquatic vegetation, a deeply incised channel, and some undercut banks. Native Owens Sucker and non-native Brown and Rainbow Trout are abundant here as well.

Layton Spring Creek (Layton Springs)— Introgressed, stream

Layton spring is a coldwater spring near the west shore of Lake Crowley. From its headspring, it feeds a short (< ¼ mile), narrow stream with gravel and cobble substrate that flows into Lake Crowley. Tui Chub are found in slower reaches of the stream. Cutthroat, Rainbow, and Brown Trout can be found throughout the stream. Sacramento Perch, presumably migrants from Crowley Lake, can be found in the very lower reaches of the stream as well.

Twin Lakes Mammoth— Introgressed, lake

Twin Lakes are a series of two montane lakes near (upstream of) the town of Mammoth Lakes in Mono County, CA. The two lakes are connected by a very short stream section that allows fish to move freely between them. The upper lake is fed by a cascading stream from a small pond below lakes Mamie and Mary. The lakes are surrounded by coniferous forest. On the west side of lake there are large boulder habitats from rock slides on a steep sloping face. At lower water levels, much of the lakes become shallow and densely vegetated. Tui Chub can be found cruising the vegetated areas of the lakes. The lakes are popular recreational fishing destinations for introduced trout.

Crowley Lake— Introgressed, lake

Crowley Lake is a reservoir formed by a dam on the upper Owens River near the town of Mammoth Lake in Mono County, CA. It is eutrophic and turbid. Substrates are predominantly silt and sand, though there are also areas with large boulders and other structure. In addition to Tui Chub, the lake supports a recreational fishery for non-native brown, rainbow, and cutthroat trout. Sacramento perch, which are native to California but not the Owens Basin, are also abundant in the reservoir. Adult Tui Chub are found primarily in areas near dense beds of aquatic vegetation, but can also be found near marinas and in bays, especially during late spring. Crowley Lake is primarily fed by the Owens River, but a number of smaller creeks carrying snowmelt or spring water flow into the creek as well. We sampled along the eastern shore of the reservoir near the Layton Spring Creek inlet.

Warm Lake— Introgressed, lake

Warm Lake is a shallow, alkaline, spring-fed lake in Long Valley near the town of Mammoth Lakes in Mono County, CA. It is connected by a very short stream with Big Alkali Lake. As the name implies, the lake receives geothermal input which contributes to its anomalous water chemistry and warm temperatures. The lake has predominantly fine silt and muddy substrate. Tui Chub appear to be the only fish in the lake.

June Lake— Introgressed, lake

June Lake is a cold, oligotrophic montane lake near the town of June Lake in Mono County, CA. June Lake sits on the southern rim of the Mono Basin, and is outside the historical distribution of Owens Tui Chub. The source and mechanism of introduction of Tui Chub to June Lake is unknown. The lake also hosts brown, rainbow, and Lahontan cutthroat trout, which were introduced for recreational fishing, as well as threespine stickleback and suckers. Tui Chub congregate in shallow littoral habitats, including near docks and marinas.

References:

Galicia D, Leunda PM, Miranda R, et al (2015) Morphometric Contribution to the Detection of Introgressive Hybridization in the Endangered Owens Tui Chub in California. Trans Am Fish Soc 144:431–442. https://doi.org/10.1080/00028487.2014.996669
