# Supplementary material for: Introgressive hybridization erodes morphological divergence between lentic and lotic habitats in an endangered minnow
Source: Ecol Evol. 2021 Sep 15;11(19):13593–600. doi: 10.1002/ece3.8086 (PMC8495819; doi:10.1002/ece3.8086)
Supplement: Supplementary file 2 — Appendix S2 [file ECE3-11-13593-s001.docx]

**Appendix S2**

**Results Tables**

**Table A2-1.** Linear model results for GPA-aligned landmark coordinates of ventral body shape. Significant predictors are bolded.

|  | ***Ventral Body Shape*** | | | | | | | |
| --- | --- | --- | --- | --- | --- | --- | --- | --- |
| Introgressed | *Independent Variable* | *df* | *SS* | *MS* | *R2* | *F* | *Z* | *P-Value* |
|  | **Centroid size (log-transformed)** | **1** | **0.15** | **0.15** | **0.16** | **76.11** | **7.16** | **<0.001** |
|  | Habitat type | 1 | 0.01 | 0.01 | 0.01 | 0.57 | -0.72 | 0.765 |
|  | **Habitat type:site** | **7** | **0.17** | **0.02** | **0.17** | **11.98** | **10.96** | **<0.001** |
|  | Residuals | 315 | 0.62 | 0.00 | 0.65 |  |  |  |
|  | Total | 324 | 0.95 |  |  |  |  |  |
| Parent subspecies | *Independent Variable* | *df* | *SS* | *MS* | *R2* | *F* | *Z* | *P-Value* |
|  | **Centroid size (log-transformed)** | **1** | **0.05** | **0.05** | **0.09** | **21.28** | **4.77** | **<0.001** |
|  | **Subspecies** | **1** | **0.08** | **0.08** | **0.16** | **39.00** | **5.84** | **<0.001** |
|  | **Habitat type** | **1** | **0.11** | **0.11** | **0.20** | **49.81** | **6.51** | **<0.001** |
|  | **Subspecies:Habitat type** | **1** | **0.01** | **0.01** | **0.03** | **6.92** | **3.88** | **<0.001** |
|  | Residuals | 128 | 0.27 | 0.00 | 0.52 |  |  |  |
|  | Total | 132 | 0.52 |  |  |  |  |  |

**Table A2-2.** Linear model results for GPA-aligned landmark coordinates of lateral body shape. Significant predictors are bolded.

|  | ***Lateral Body Shape*** |  |  |  |  |  |  |  |
| --- | --- | --- | --- | --- | --- | --- | --- | --- |
| Introgressed | *Independent Variable* | *df* | *SS* | *MS* | *R2* | *F* | *Z* | *P-Value* |
|  | **Centroid size (log-transformed)** | **1** | **0.05** | **0.05** | **0.08** | **35.24** | **7.62** | **<0.001** |
|  | Habitat type | 1 | 0.01 | 0.01 | 0.02 | 0.89 | -0.14 | 0.559 |
|  | **Habitat type:site** | **7** | **0.10** | **0.01** | **0.16** | **9.43** | **12.23** | **<0.001** |
|  | Residuals | 315 | 0.48 | 0.00 | 0.74 |  |  |  |
|  | Total | 324 | 0.65 |  |  |  |  |  |
| Parent subspecies | *Independent Variable* | *df* | *SS* | *MS* | *R2* | *F* | *Z* | *P-Value* |
|  | **Centroid size (log-transformed)** | **1** | **0.03** | **0.03** | **0.09** | **18.99** | **6.25** | **<0.001** |
|  | **Subspecies** | **1** | **0.03** | **0.03** | **0.10** | **21.27** | **6.38** | **<0.001** |
|  | **Habitat type** | **1** | **0.03** | **0.03** | **0.12** | **24.42** | **7.14** | **<0.001** |
|  | **Subspecies:Habitat type** | **1** | **0.01** | **0.01** | **0.05** | **10.68** | **5.64** | **<0.001** |
|  | Residuals | 128 | 0.17 | 0.00 | 0.63 |  |  |  |
|  | Total | 132 | 0.27 |  |  |  |  |  |

**Table A2-3.** Linear model results for GPA-aligned landmark coordinates of dorsal body shape. Significant predictors are bolded.

|  | ***Dorsal Body Shape*** | | | | | | | |
| --- | --- | --- | --- | --- | --- | --- | --- | --- |
| Introgressed | *Independent Variable* | *df* | *SS* | *MS* | *R2* | *F* | *Z* | *P-Value* |
|  | **Centroid size (log-transformed)** | **1** | **0.12** | **0.12** | **0.13** | **58.97** | **5.56** | **<0.001** |
|  | Habitat type | 1 | 0.03 | 0.03 | 0.03 | 1.94 | 1.05 | 0.152 |
|  | **Habitat type:site** | **7** | **0.12** | **0.02** | **0.12** | **7.94** | **6.64** | **<0.001** |
|  | Residuals | 315 | 0.67 | 0.00 | 0.71 |  |  |  |
|  | Total | 324 | 0.94 |  |  |  |  |  |
| Parent subspecies | *Independent Variable* | *df* | *SS* | *MS* | *R2* | *F* | *Z* | *P-Value* |
|  | **Centroid size (log-transformed)** | **1** | **0.03** | **0.03** | **0.12** | **24.37** | **4.47** | **<0.001** |
|  | **Subspecies** | **1** | **0.02** | **0.02** | **0.06** | **12.82** | **3.65** | **<0.001** |
|  | **Habitat type** | **1** | **0.05** | **0.05** | **0.17** | **35.32** | **4.94** | **<0.001** |
|  | **Subspecies:Habitat type** | **1** | **0.01** | **0.01** | **0.03** | **5.20** | **2.56** | **<0.005** |
|  | Residuals | 128 | 0.17 | 0.00 | 0.62 |  |  |  |
|  | Total | 132 | 0.27 |  |  |  |  |  |

**Table A2-4.** Results from linear (mixed) models of caudal fin aspect ratio.

|  | ***Caudal Fin Aspect Ratio*** | | | | | |
| --- | --- | --- | --- | --- | --- | --- |
| Introgressed | *Independent Variable* | *df*  *(Satterthwaite)* | *Estimate* | *Std. Error* | *t-value* | *P-Value* |
|  | Intercept | 64.09 | 0.33 | 0.18 | 1.85 | 0.069 |
|  | **Standard length (log-transformed)** | **94.57** | **0.27** | **0.04** | **6.91** | **<<0.001** |
|  | Habitat type | 5.02 | 0.08 | 0.06 | 1.39 | 0.223 |
| Parent subspecies | *Independent Variable* | *df* | *Estimate* | *Std. Error* | *t-value* | *P-Value* |
|  | Standard length (log-transformed) | 1 | 0.61 | 0.61 | 3.88 | 0.051 |
|  | Habitat type | 1 | 1.50 | 1.50 | 9.54 | <0.01 |
|  | Subspecies | 1 | 1.31 | 1.31 | 8.36 | <0.05 |
|  | Subspecies:Habitat type | 1 | 0.17 | 0.17 | 1.06 | 0.305 |
|  | Residuals | 119 | 18.72 | 0.16 |  |  |

**Table A2-5.** Morphological disparity measured as procrustes variance of landmark coordinates combined for dorsal, lateral, and ventral perspectives. Colors show putative subspecies (Owens = Blue, Lahontan = Yellow, Introgressed = Green).

| **Site** | **Abbreviation** | **Habitat Type** | **Procrustes Variance** |
| --- | --- | --- | --- |
| Crowley Lake | CL | Lake | 0.00197 |
| East Walker River | EWR | Stream | 0.00267 |
| Hot Creek | HC | Stream | 0.00289 |
| June Lake | JL | Lake | 0.00279 |
| Layton Springs | LS | Stream | 0.00321 |
| Little Hot Creek | LHC | Stream | 0.00329 |
| Mammoth Creek | MC | Stream | 0.00172 |
| McNally Canal | MNC | Stream | 0.00192 |
| Owens River | OR | Stream | 0.00256 |
| Twin Lakes (Mammoth) | TLM | Lake | 0.00224 |
| Twin Lakes (Bridgeport) | TLB | Lake | 0.00368 |
| Warm Lake | WL | Lake | 0.00247 |
| White Mountain Research Station | WMRS | Lake | 0.00509 |

**Table A2-6.** P-values from pairwise comparisons by permutation of morphological disparity, measured as procrustes variance. Pure Owens populations are shaded blue, Lahontan are yellow, and introgressed are green. Significant differences (α = 0.05) are bold. Site abbreviations are defined in Table A2-5.

| **CL** | 1 |  |  |  |  |  |  |  |  |  |  |  |  |
| --- | --- | --- | --- | --- | --- | --- | --- | --- | --- | --- | --- | --- | --- |
| **EWR** | 0.186 | 1 |  |  |  |  |  |  |  |  |  |  |  |
| **HC** | 0.064 | 0.681 | 1 |  |  |  |  |  |  |  |  |  |  |
| **JL** | 0.120 | 0.822 | 0.850 | 1 |  |  |  |  |  |  |  |  |  |
| **LS** | **0.020** | 0.331 | 0.538 | 0.448 | 1 |  |  |  |  |  |  |  |  |
| **LHC** | **0.014** | 0.278 | 0.449 | 0.378 | 0.87 | 1 |  |  |  |  |  |  |  |
| **MC** | 0.634 | 0.102 | **0.032** | 0.064 | **0.01** | **0.008** | 1 |  |  |  |  |  |  |
| **MNC** | 0.926 | 0.172 | **0.059** | 0.102 | **0.02** | **0.013** | 0.701 | 1 |  |  |  |  |  |
| **OR** | 0.235 | 0.848 | 0.516 | 0.662 | 0.23 | 0.185 | 0.131 | 0.218 | 1 |  |  |  |  |
| **TLM** | 0.625 | 0.460 | 0.244 | 0.342 | **0.10** | 0.080 | 0.383 | 0.567 | 0.563 | 1 |  |  |  |
| **TLB** | **0.001** | 0.074 | 0.131 | 0.116 | 0.37 | 0.479 | **0.001** | **0.001** | **0.041** | **0.016** | 1 |  |  |
| **WL** | 0.294 | 0.700 | 0.387 | 0.528 | 0.14 | 0.122 | 0.155 | **0.260** | 0.849 | 0.669 | **0.018** | 1 |  |
| **WMRS** | **0.000** | **<0.001** | **<0.001** | **<0.001** | **<0.001** | **<0.001** | **<0.001** | **<0.001** | **<0.001** | **<0.001** | **0.010** | **<0.001** | 1 |
| **Site** | **CL** | **EWR** | **HC** | **JL** | **LS** | **LHC** | **MC** | **MNC** | **OR** | **TLM** | **TLB** | **WL** | **WMRS** |
